# Characteristics of Fournier gangrene and evaluation of the effects of negative-pressure wound therapy

**DOI:** 10.3389/fsurg.2022.1075968

**Published:** 2023-01-06

**Authors:** Rui He, Xiangyan Li, Kun Xie, Bing Wen, Xin Qi

**Affiliations:** ^1^ Department of Plastic Surgery and Burn, Peking University First Hospital, Beijing, China; ^2^ Department of Anti-Infection, Institute of Clinical Pharmacology, Peking University First Hospital, Beijing, China

**Keywords:** Fournier gangrene, negative-pressure wound therapy, necrotizing fasciitis, microbiology, treatment

## Abstract

Fournier gangrene (FG) is a life-threatening disease affecting the soft tissues of the genital, perineal, and perianal regions. This retrospective study aimed to summarize the characteristics of FG and evaluate the effects of negative-pressure wound therapy (NPWT). We analyzed clinical data of 36 patients with FG admitted to our department. Thirty-four cases had perianal and external genital infections, and the other two had secondary infection of the urinary fistula after trauma and retroperitoneal abscess, respectively. Monomicrobial, polymicrobial, culture-negative, and fungal infections were identified in 16, 17, 2, and 1 cases, respectively. *Escherichia coli, Enterococcus faecalis, Enterococcus faecium, Klebsiella pneumoniae*, and *Staphylococcus haemolyticus* were the most common pathogens. The mortality rate was 8%. Twenty-seven and nine patients were treated with NPWT (group A) and conventional dressing (group B), respectively. The length of stay was 38.0 ± 16.1 and 51.0 ± 17.3 days, number of operations were 3 (3,6) and 13 (4,17), and wound healing times were 39.2 ± 18.1 and 66.5 ± 17.1 days in groups A and B, respectively. Taken together, clinicians should always consider the possibility of perianal or external genital infections progressing to FG in the daily work, especially for patients with diabetes mellitus. *Enterobacteriaceae, Enterococcus*, and *Staphylococcus haemolyticus* are the most common causative pathogens, and NPWT is an effective adjuvant therapy for wound management with fewer operations and a shorter wound healing time.

## 1. Introduction

Fournier's gangrene (FG) is a specific form of necrotizing fasciitis that involves the soft tissues of the genital, perineal, and perianal regions. It is an uncommon disease that can affect people of any age and rapidly progress to a life-threatening stage. The incidence is reported to be 1.6 cases per 100,000 men per year and remains steady after the age of 50 at 3.3 cases per 100,000 men (1). Early diagnosis and emergency intervention are mandatory owing to the rapid rate of fascial necrosis. However, non-specific symptoms at the early stage and lack of awareness among clinicians may delay diagnosis, hence, leading to a miss of the best time for intervention. As the infection progresses, typical symptoms may present, such as blistering, purulent discharge, crepitation, or necrosis, accompanied by systemic inflammatory response syndrome, sepsis, shock, or multiple organ dysfunction syndrome (MODS), leading to death. The infection can spread to distal regions, such as the abdominal wall, ischiorectal fossa, hip, and thigh through the fascial plane. Currently, the mortality remains at 20%–30% (2, 3). Negative-pressure wound therapy (NPWT) is an effective wound management technique that is widely used for almost all types of wounds. This study aimed to summarize the characteristics of FG and evaluate the effects of NPWT.

## 2. Materials and methods

This retrospective case-control study enrolled patients with FG treated at our department between May 2010 and June 2022. Data were obtained from medical records. The diagnosis was based on clinical findings (necrosis of the skin and/or fascia, gray discolored fascia, “dishwater fluid,” lack of bleeding, and tissue resistance of the fascial layer). The extent of infection that limited itself to the genital, perineal, or perianal regions was considered as “localized;” otherwise it was considered as “diffused”. Patients were divided into two groups based on wound management: group A comprised patients treated with NPWT and group B comprised patients treated with conventional dressing. This study was approved by the Institutional Human Investigations Committee.

### 2.1. Treatment approaches

#### 2.1.1. Anti-infection treatment

Broad-spectrum anti-infection treatment comprising carbapenem and vancomycin was empirically administered upon diagnosis of FG and subsequently modified based on the culture results. The duration of anti-infection treatment required comprehensive consideration of systemic signs and symptoms, local signs of infection, and laboratory test results.

#### 2.1.2. Surgical treatment

Emergency surgical debridement was performed within 24 h after admission. An incision was made in the area of sclerosis, erythema, ulceration, or necrosis. The necrotic tissue was removed, drainage was ensured, and thorough dissection along the fascial plane was performed to explore the extent of the infection until viable tissue was identified. Samples for culture were routinely obtained. Digital rectal examination was routinely performed to confirm the presence of anal fistula. Colostomy was performed for patients with anal fistulas or severe perianal involvement. Suprapubic cystostomy was performed for patients with urethral fistulas.

**Figure 1:**
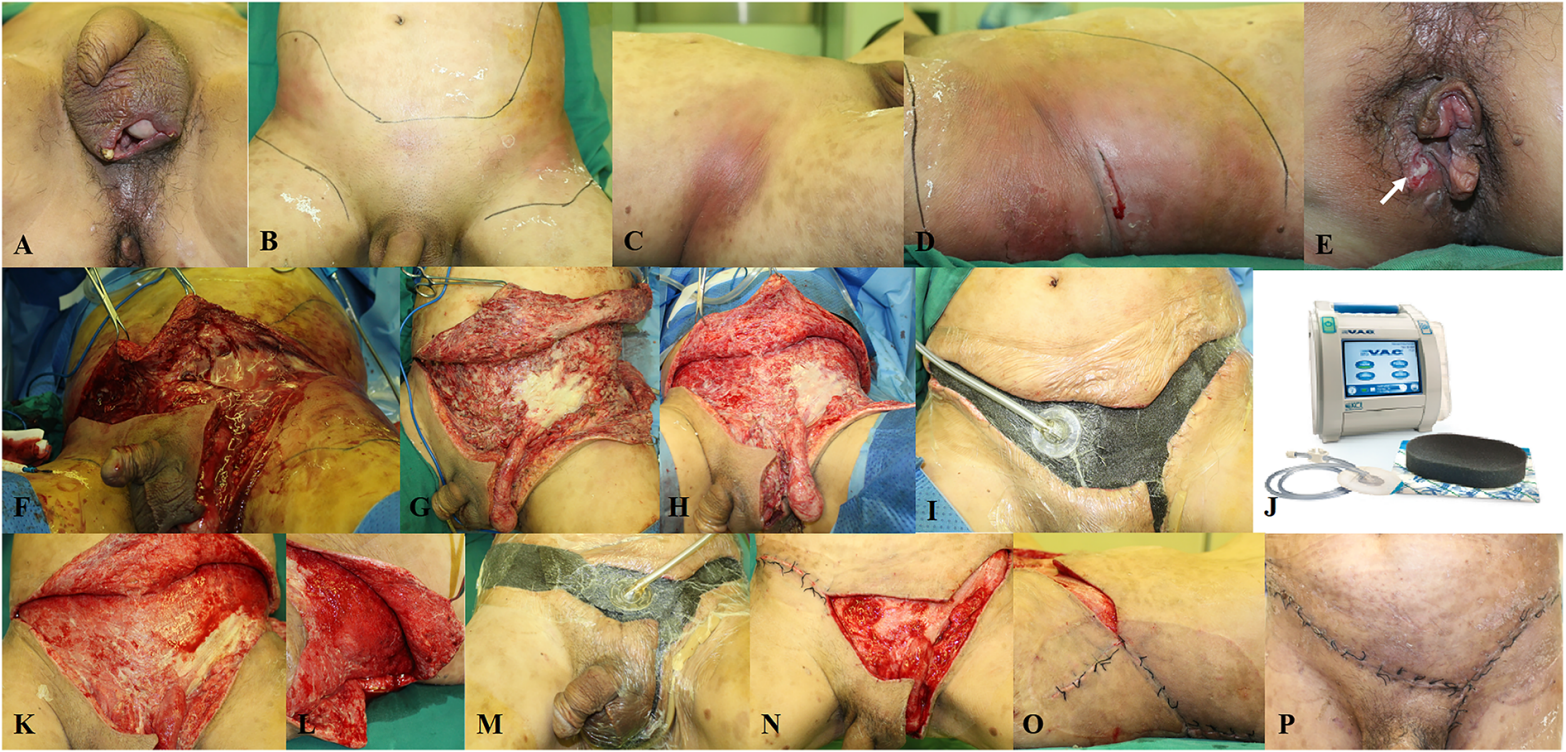
A representative case of a 50-year-old patient diagnosed with fournier gangrene and transferred to our department. (**A–D**) Despite incision and drainage of the scrotum before admission, the infection spread to the lower and lateral abdominal wall. (**E**) The etiology is the perianal abscess (white arrow). (**F**) Emergency surgical debridement is performed. (**G**) Appearance after debridement. (**H**) Appearance before the second debridement. (**I**) NPWT is applied during the third debridement. (**J**) The V.A.C. system used in our department. (**K,L**) Appearance before the fourth debridement. (**M**) Part of the wound is closed and NPWT is continued after the fourth debridement. (**N,O**) Appearance before the fifth debridement; the rest of the wound is closed. (**P**) Appearance before discharge.

After the initial debridement, conventional dressing or NPWT was used for postoperative wound management based on patients' choice. Dressings were changed when the wound became wet for the conventional method or every 5–7 days when NPWT was applied. Negative pressure was applied in continuous mode with the suction level of −125 mmHg. A series of debridement procedures were performed if residual necrotic tissue was present. The wound reconstruction method depended on the location and extent of the defect (Figure 1).

#### 2.1.3. Supportive treatment

Nutritional support, glucose control, fluid and electrolyte balance, blood product transfusion, and comorbidity control were also important treatments for FG. Patients with hemodynamic instability, septic shock, or MODS were transferred to the intensive care unit.

### 2.2. Data collection

Medical record data including demographic data, initial symptoms, physical examination findings, vital signs, treatment before admission, comorbidities, laboratory tests, microbiological results, American Society of Anesthesiologists score, number of operations, extent of infection, wound coverage, length of stay, and wound healing time were collected. Laboratory risk indicators for necrotizing fasciitis (LRINEC) (4) were also calculated.

### 2.3. Statistical analysis

Statistical analyses were performed using SPSS software version 22. Continuous parametric variables are shown as means with standard deviations (SDs) and continuous nonparametric variables, as medians with interquartile ranges (IQRs). All continuous variables were subjected to the Kolmogorov–Smirnov test. Normally distributed variables were analyzed using an independent-sample *t*-test. Otherwise, the variables were compared using the Mann–Whitney *U* test. Categorical variables were analyzed using the Pearson's *χ*^2^ test (Fisher's exact test, as appropriate). *P* < 0.05 was considered to be statistically significant.

## 3. Results

Thirty-six patients were enrolled in the study. Patient characteristics and diseases are shown in Table 1. Diabetes mellitus (DM) was the most common comorbidity (53%). Twenty-eight patients were transferred to our department from other hospitals, 12 of whom had undergone incision and drainage. The predominant etiologies were perianal (64%) and external genital (31%) infections. The infection had spread to the distal part beyond the genital, perineal, or perianal regions in 24 cases, including the lower or lateral abdominal wall, inguinal region, thigh, ischiorectal fossa, and hip.

**Table 1:** Characteristics of patients and diseases.

Parameters	Values
Demographic	
Age, years	55.9 ± 11.6
Males, *n*	33
BMI, kg/m^2^	25.8 ± 3.8
Comorbidities	
Diabetes mellitus, *n*	19
Hypertension, *n*	17
Chronic kidney disease, *n*	5
Cerebrovascular disease. *n*	3
Coronary heart disease, *n*	3
Malignancy, *n*	2
Etiologies	
Perianal infections, *n*	23
External genital infections, *n*	11
Traumatic urinary fistula, *n*	1
Retroperitoneal abscess, *n*	1
Extent of infection	
Localized, *n*	12
Diffused, *n*	24
Types of infections	
Polymicrobial infection, *n*	17
Monomicrobial infection, *n*	16
Fungal infection, *n*	1
Wound reconstruction	
Delayed primary closure, *n*	16
Secondary intension, *n*	2
STSG, *n*	15
ICU, *n*	10
Mortality, *n*	3

BMI, body mass index; STSG, split-thickness skin graft; ICU, intensive care unit.

Pathogens were identified in 34 (95%) patients. The details of the 49 bacterial strains cultured were listed in Table 2. Enterobacteriaceae, *Enterococcus*, and *Staphylococcus haemolyticus* were the most common causative pathogens. The resistance rate of gram-negative bacilli to third-generation cephalosporins was 35%. Vancomycin-resistant gram-positive bacteria were not found, but all *S. haemolyticus* isolates were methicillin resistant.

**Table 2:** Microbiological results.

Pathogens	Number
Gram-positive	
*Enterococcus faecalis*	8
*Enterococcus faecium*	6
*Staphylococcus haemolyticus*	5
*Enterococcus avium*	2
*Enterococcus gallinarum*	1
*Staphylococcus lugdunensis*	1
*Streptococcus pyogenes*	1
*Streptococcus agalactis*	1
*Streptococcus oligoacidus*	1
Gram-negative	
*Escherichia coli*	13
*Klebsiella pneumoniae*	6
*Pseudomonas aeruginosa*	2
*Proteus mirabilis*	1
*Proteus vulgaris*	1

Colostomy and suprapubic cystostomy was performed in 10 and 2 patients, respectively. The mortality rate was 8%. Delayed primary closure, secondary intention, or split-thickness skin grafts (STSG) were applied for wound reconstruction.

### 3.1. Comparison between the two groups

Twenty-seven and nine patients received NPWT (group A) and conventional dressing treatment (group B), respectively. There were no significant differences in the baseline clinical characteristics between the two groups (Table 2). However, the number of operations in group A was significantly lower than that in group B, and the wound healing time in group A was significantly shorter than that in group B (Table 3).

**Table 3:** Comparison of clinical characteristics and outcomes between two groups.

Parameters	Group A (*n* = 27)	Group B (*n* = 9)	*P*
Demographic			
Age	55.9 ± 11.7	55.7 ± 11.9	0.955
Males	25	8	1.000
BMI, kg/m^2^	26.18 ± 3.77	24.71 ± 3.72	0.320
Comorbidities			
Diabetes mellitus	14	5	1.000
Hypertension	15	2	0.128
ASA			0.063
2	13	4	
3	11	1	
4	3	4	
Extent of infection			0.432
Localized	12	2	
Diffused	15	7	
LRINEC	6 (3,9)	9 (4.5,10.0)	0.127
Clinical outcomes			
Length of stay, days	38.0 ± 16.1	51.0 ± 17.3	0.089
Number of operation	3 (3, 6)	13 (4, 17)	**0.026**
Wound healing time, days	39.2 ± 18.1	66.5 ± 17.1	**0.002**

BMI, body mass index; ASA, american society of anesthesiologists; LRINEC, laboratory risk indicator for necrotizing fasciitis. *P-*values in bold are statistically significant.

## 4. Discussion

FG is a fulminant and life-threatening disease that requires timely and standardized intervention. The initial symptoms vary; therefore, FG is easily misdiagnosed as cellulitis or erysipelas. Once typical symptoms appear, the infection is usually severe or has already spread to the distal regions. Pathogens play an important role in the development of FG (5). We enrolled a relatively large number of patients with FG, summarized their characteristics, provided the spectrum of pathogens, shared treatment experiences, and evaluated the effects of NPWT.

DM is one of the main predisposing factors, apart from immunosuppression, malignancy, and chronic steroid use (5). Local intravenous drug injection can also be the predisposing factor in rare cases (6). Concordant with previous studies, 53% of patients in the present study had DM. A meta-analysis showed that mortality due to FG is associated with DM (7). In the present study, the prevalence of DM in patients who died (67%) was higher than that in patients who survived (52%); however, the relatively small number of patients who died precludes statistical conclusions from being drawn. Perianal and external genital infections were the predominant etiologies, and the infection had spread to the distal parts in most cases (67%). This finding was consistent with the anatomical features and pathogenesis of FG. Perianal sources of infection can spread to the perineal fascia through perianal space, and fascial systems of the penis, scrotum, perineum, and abdominal wall are all connected. Therefore, perianal abscess may involve the Colles fascia, and the infection may spread to the penis and scrotum *via* Buck's and Dartos fascia, or to the anterior abdominal wall *via* Scarpa's fascia, or vice versa. Therefore, to avoid misdiagnosis or delayed diagnosis, clinicians should always consider the possibility of perianal or external genital infections progressing to FG in the daily work, especially for patients with DM. Patients are recommended to routinely undergo LRINEC scoring, closely observe the wound, and maintain timely communication.

An early diagnosis of FG is challenging, but the principles of treatment are clear and include aggressive debridement, anti-infection treatment, and supportive treatment (5, 7). Furthermore, the principles of anti-infection treatment depend on empirical broad-spectrum antibiotic coverage before pathogen identification. Therefore, it is imperative to understand the characteristics of these pathogens. Some studies suggested polymicrobial infection as the predominant type (5, 8, 9), but another study identified predominant incidence of monomicrobial gram-negative organisms in southwestern China (10). Nevertheless, *Escherichia coli* is the most frequently isolated microorganism (2, 9, 10). The generally accepted and recommended empirical antibiotic therapy includes aminoglycosides, metronidazole or clindamycin, and third-generation cephalosporin (5, 9). In the present study, the incidence of monomicrobial infections was approximately equal to that of polymicrobial infections. *Escherichia coli* and *Klebsiella pneumoniae* were the most common gram-negative, causative pathogens, whereas *Enterococcus faecalis*, *Enterococcus faecium*, and *Staphylococcus haemolyticus* were the most common gram-positive, causative pathogens. These findings are consistent with the predominant etiology of FG. The proportion of resistant bacteria was high; the resistance rate of gram-negative bacilli to third-generation cephalosporins was 35%, and all cases with *Staphylococcus haemolyticus* were methicillin resistant. Therefore, considering the drug resistance and severity of FG, carbapenem and vancomycin were used as empirical antibiotic therapies at our department. We believe that empirical antibiotic therapy in different regions should be consistent with the characteristics of the pathogens. Furthermore, although fungal infections have rarely been reported in the literature, fungal smears should be indicated to clarify the possibilities of fungal infections.

As a series of debridement procedures are normally required, an appropriate method for wound management should be applied. For conventional dressing treatment, frequent dressing changes, difficulty in timely and adequate control of exudate, and limited mobility are disadvantages. However, NPWT can create a controlled and closed negative-pressure environment. By continuously removing the wound exudate, reducing the wound area, increasing blood perfusion, and stimulating angiogenesis, it can effectively promote wound healing (11). Owing to these advantages, NPWT has been widely used to treat various types of wounds. In the present study, NPWT significantly reduced the number of operations and shortened wound healing time than conventional dressing treatment. Moreover, NPWT can effectively fix grafted skin on the surface of irregular wounds, such as the penis or scrotum. Patients can carry the device while moving in the ward, ensuring increased compliance to the treatment. However, NPWT cannot replace surgical debridement as a wound management technique as inappropriate use can aggravate infection. Colostomy is recommended for patients with anal fistulas or severe perianal involvement, making NPWT feasible. Hyperbaric oxygen therapy (HBOT) is also an effective adjuvant therapy for FG, and NPWT combined with HBOT shows advanced wound healing with a high efficiency rate (12). However, the main reason hampering the use of HBOT is the poor availability of the devices.

The present study has some limitations. First, as this was a single-center retrospective study, the applicability of the findings may be limited. However, the cohort size was relatively larger than that of other single-center studies. Thus, for the possible differences in the pathogenic spectrum, we also proposed relevant principles. Second, as several patients were transferred from other hospitals, some initial laboratory parameters were unavailable and previous treatment may have caused changes in the laboratory test results. However, owing to the low incidence of FG and difference in medical capacity, this phenomenon cannot be avoided.

## 5. Conclusion

Clinicians should always consider the possibility of perianal or external genital infections progressing to FG in the daily work, especially for patients with DM. Enterobacteriaceae, *Enterococcus*, and *Staphylococcus haemolyticus* were the most common causative agents. NPWT is an effective adjuvant therapy for wound management, with fewer operations and shorter wound healing times.

## Data Availability

The raw data supporting the conclusions of this article will be made available by the authors, without undue reservation.
